# Supplementary material for: Genomic characterization of a nematode tolerance locus in sugar beet
Source: BMC Genomics. 2023 Dec 6;24:748. doi: 10.1186/s12864-023-09823-2 (PMC10702057; doi:10.1186/s12864-023-09823-2)
Supplement: Supplementary file 7 — Supplementary Material 7 [file 12864_2023_9823_MOESM7_ESM.pdf]

Supplementary Material - Legends

Additional file S1: S1A: Genomic positions of the new set of 187 markers designed based on data for Strube U2Bv. In the first column, the name of the marker is shown, followed by the chromosome and genomic position for each assembly (2320BvONT v1.0 and U2BvONT v1.0). **S1B:** Overrepresentation analysis (GO terms) of the AtNLP7 targets identified by Alvarez et al., 2020. **S1C:** Percent identity matrices for the BvNLP7 genes. **S1D:** List of all significantly differentially expressed genes for the comparison all tolerant vs all susceptible. **S1E:** List of all significantly differentially expressed genes for the comparison SUS3 treated vs BR12 treated. **S1F:** List of all significantly differentially expressed genes for the comparison U1Bv treated vs U2Bv treated. **S1G:** List of functionally annotated DEGs for the comparison all tolerant vs all susceptible. **S1H:** List of functionally annotated DEGs for the comparison SUS3 treated vs BR12 treated. **S1I:** List of functionally annotated DEGs for the comparison U1Bv treated vs U2Bv treated.

Additional file S2: S2A: Histogram of adjusted cyst counts (SN) data per family. **S2B:** Structural rearrangements between 2320BvONT and U2BvONT as identified with SyRI. **S2C:** Delta allele frequency plots for 10 SNP windows of all nine U2BvONT pseudochromosomes. **S2D:** Marker information/primer sequences used for delimitation of the potential NT locus and graphical genotyping with flanking and co-segregating markers. **S2E:** Visualization of key polymorphisms at conserved positions of Arabidopsis NLP7. **S2F:** Principal component analysis for all samples of the RNA-Seq infection experiment. **S2G:** Availability and composition of datasets generated. **S2H:** Detailed methods for i) calculation of the delta allele frequencies and interval detection, ii) ONT sequencing, iii) ONT assembly, and iv) gene prediction and

functional annotation. **S2I:** Overview of the RNA-Seq samples in the infection experiment. **S2J:** List of all RNA-Seq datasets incorporated as hints for the gene prediction including two newly submitted datasets. **S2K:** Dot plot heatmap of the closed gap region in the initial Strube U2Bv assembly.

Additional file S3: Multiple sequence alignment of coding sequences of all *BvNLP7* genes.

Additional file S4: Multiple sequence alignment of *A. thaliana* NLP7 and U2BvONT and 2320BvONT rycp aa sequences.

Additional file S5: Multiple sequence alignment of aa sequences encoded by the *BvNLP7* genes (without rycp).

Additional file S6: Multiple sequence alignment of aa sequences encoded by all BvNLP7 genes.
